# Old and New Insights into *Sporothrix schenckii* Complex Biology and Identification

**DOI:** 10.3390/pathogens11030297

**Published:** 2022-02-25

**Authors:** Elena De Carolis, Brunella Posteraro, Maurizio Sanguinetti

**Affiliations:** 1 Dipartimento di Scienze di Laboratorio e Infettivologiche, Fondazione Policlinico Universitario A. Gemelli IRCCS, 00168 Rome, Italy; maurizio.sanguinetti@policlinicogemelli.it; 2 Dipartimento di Scienze Biotecnologiche di Base, Cliniche Intensivologiche e Perioperatorie, Università Cattolica del Sacro Cuore, 00168 Rome, Italy; brunella.posteraro@policlinicogemelli.it

**Keywords:** *Sporothrix schenckii* complex, closely related species, morphological shift, diagnostic methods

## Abstract

*Sporothrix schenckii* is a worldwide-distributed thermally dimorphic fungus, which usually causes a subacute to chronic infection through traumatic implantation or inoculation of its infectious propagules. The fungus encompasses a group of phylogenetically closely related species, thus named the *S. schenckii* complex, of which *S. schenckii* *sensu stricto* and *S. brasiliensis* are main causative species of sporotrichosis. Owing to a multifaceted molecular dynamic, the *S. schenckii* complex can switch between the mycelium and the yeast form. This characteristic along with a varying cell wall composition account for significant species-specific differences in the host range, virulence, and susceptibility to antifungal drugs. While culture remains the gold standard to diagnose sporotrichosis, polymerase chain reaction (PCR) or matrix-assisted laser desorption ionization time-of-flight (MALDI-TOF) mass spectrometry-based methods have become an essential for accurate species identification in many clinical laboratories. If directly applied on tissue samples, molecular methods are helpful to improve both sensitivity of and time to the etiological diagnosis of sporotrichosis. This mini-review aims to put together the old and new knowledge on the *S. schenckii* complex biology and identification, with particular emphasis on the laboratory diagnosis-related aspects of disease.

## 1. Introduction

Originally known as the only causative agent of human and animal sporotrichosis [1], the thermally dimorphic *Sporothrix schenckii* (*sensu lato*) really consists of phylogenetically closely related species, which are embedded in the order Ophiostomatales, class Pyrenomycetes, division Ascomycota within the Fungi kingdom [2]. According to Marimon et al.’s studies [3,4], the *S. schenckii* complex encompasses four clinically relevant species (*S. schenckii sensu stricto*, *S. brasiliensis*, *S. globosa*, and *S. luriei*) and two species (*S. mexicana* and *S. albicans*) occasionally isolated from humans. Although *Sporothrix schenckii* (*sensu lato*) is regarded as a cosmopolitan fungus, most sporotrichosis cases occur in tropical and subtropical areas from Latin America, Africa, and Asia [1,5]. Whereas *S. schenckii sensu stricto* and *S. globosa* are distributed throughout the world, *S. brasiliensis* has emerged in the Rio de Janerio state of and is restricted to Brazil [1,5]. Apart from the geographical distribution, significant differences in the host range, virulence, and antifungal drug susceptibility have been observed among the four mentioned species [6,7,8].

Sporotrichosis is a subacute or chronic implantation (formerly subcutaneous) mycosis that follows the traumatic inoculation of infectious propagules (conidia from the mycelial phase), released from the fungus in the environment, into the skin, mucosal, or osteoarticular body sites [9]. Consistently, since its first description by Schenck in 1898, the disease has been associated with human activities (e.g., floriculture, horticulture, gardening, or hunting) that facilitate the contact with decaying plant material contaminated by the fungus, thereby the name of “rose gardener’s disease” or “rose handler’s disease” with which sporotrichosis has mainly been known. However, in addition to the sapronotic (plant) transmission that, in endemic regions, primarily regards *S. schenckii sensu stricto* and *S. globosa*, a zoonotic transmission by which *S. brasiliensis* spread through deep scratches and bites from infected felines (cats) has been associated with more severe clinical presentations in humans [10].

If not limited to skin (cutaneous-lymphatic or cutaneous-fixed sporotrichosis), sporotrichosis manifestations include cutaneous-disseminated, disseminated (visceral), and extracutaneous forms (pulmonary, osteoarticular, and meningeal), which are prominently diagnosed in immunocompromised (human immunodeficiency virus (HIV)-AIDS, chronic alcoholism, diabetes, hematological cancer, or transplantation) patients [10]. Primary pulmonary sporotrichosis, which results from inhalation of conidia, may lead to a disseminated disease and is often fatal [11]. In feline sporotrichosis, uniquely when compared to the infections caused by other endemic dimorphic fungi (e.g., Onygenales), transmission seems to occur directly through the fungus’s yeast phase that is present at a high burden in the infected animal’s lesions [10]. Another distinctive feature of sporotrichosis is the supposed high prevalence of animal-to-human transmission, which is consistent with the so far described largest outbreak of cat-transmitted sporotrichosis that has emerged three decades ago and continued to expand in Brazil [12], involving thousands of humans and cats. Interestingly, some of them were immunocompromised subjects affected by HIV and feline immunodeficiency virus (FIV), respectively [10]. The outbreak-causing *Sporothrix* species identified was *S. brasiliensis* [13] that, according to a phylogenomic analysis conducted in 2014 by Teixeira et al. [14], diverged from *S. schenckii sensu stricto* about 3.8–4.9 million years ago, which suggests that a recent event of speciation has occurred in the genus *Sporothrix*.

In this review, we summarize recently or less recently published findings on the *S. schenckii* complex, to emphasize relevant aspects of biology and identification of human and animal sporotrichosis agents.

## 2. Species-Level Identification

By means of molecular taxonomic markers as discussed below, it has become clear that the main pathogenic *Sporothrix* species cluster in a derived clade around *S. schenckii sensu stricto*, resulting in at least four “cryptic” species (i.e., species differentiated from each other only through molecular methods) within the *S. schenckii* complex [15]. As already mentioned, these species may vary in geographic distribution, host range, virulence, and antifungal susceptibility. This makes the species-level identification of etiological agents of sporotrichosis the mainstay in the laboratory diagnosis of disease [16,17].

### 2.1. Microscopy, Culture, and Immunology-Based Methods

Microscopic examination of potassium hydroxide-pretreated clinical samples obtained from skin lesions (pus and exudates), sputum, or synovial fluid from patients with sporotrichosis allows (albeit at low sensitivity) to visualize yeast forms of 2–6 µm in diameter. Despite a slight Gram-staining positivity, yeast cells are usually observed close to giant multinucleated or polymorphonuclear cells [16]. Otherwise, microscopic examination of lesion exudate in 10% formaldehyde solution allowing high-sensitivity visualization of asteroid bodies (i.e., yeasts surrounded by host immunoglobulins) may be helpful for timely diagnosis and treatment [16]. In sporotrichosis, clinical samples inoculated on mycological media (e.g., Sabouraud dextrose agar (SDA)) yields after incubation at 28 °C for 5–8 days white filamentous colonies, which convert in white to creamy yeast-like colonies when subcultured in a brain–heart infusion medium at 37 °C for 5–10 days. Subsequently, micromorphology analyses of the mycelial and yeast forms of the fungus (to observe typical features as already detailed), along with physiological and/or biochemical tests, allows accurate identification of the *S. schenckii* complex as a whole but not that of the single species within the *S. schenckii* complex [17].

Immunological diagnoses of sporotrichosis rely on the *Sporothrix*-specific antibody detection in the sera from *Sporothrix*-infected patients using agglutination and immunoenzymatic (e.g., ELISA) assays [16]. To develop one of these assays, the cell wall PMR had been fractionated by affinity chromatography with concanavalin A (Con A) and the Con A-bound fraction probed with an anti-*S. schenckii* rabbit serum. The resulting antigen, thus named *Ss*CBF (*S. schenckii* Con A-binding fraction), was specifically recognized by IgG antibodies present in patients’ clinical samples [16]. Further studies comparing the reactivity of this antigen recovered from three *S. schenckii* strains showed that the antigen isolated from strain 1099-18 provided an accurate means for the serodiagnosis of sporotrichosis [18]. Despite their high performance [19], serological tests are not useful to diagnose acute infections or past infections in immunocompromised patients [20], whereas no immunological methods described until now can differentiate the species of the *S. schenckii* complex [17].

### 2.2. Molecular Biology-Based Methods

**Figure 1:**
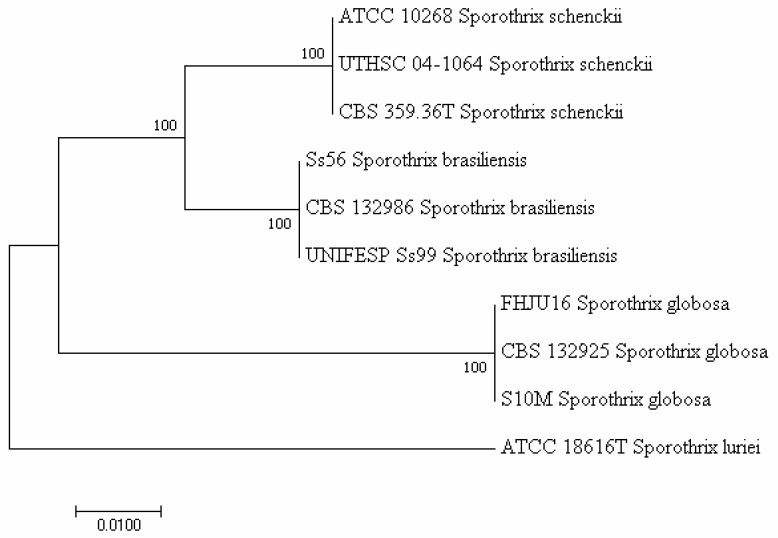
Neighbor-joining phylogenetic tree of *CAL* gene sequences of 10 strains belonging to the clinical *S. schenckii* complex clade. The evolutionary distances were computed using the Maximum Composite Likelihood method.

In 2007, Marimon et al. [4] supported the evidence raised from polymerase chain reaction (PCR)-based molecular studies, as summarized in the study by Oliveira et al. [17], that phenotypically overlapping *S. schenckii* isolates do not belong to the same species. The authors described four *S. schenckii* (*sensu stricto*)-related species (*S. brasiliensis*, *S. globosa*, *S. luriei*, and *S. mexicana*) through a combination of phenotypic and genetic features and proposed the partial calmodulin (CAL) gene as a molecular tool to differentiate (phylogenetically) closely related species. However, Zhou et al. [21] and Rodrigues et al. [22] showed that ribosomal DNA (rDNA) internal transcribed spacer (ITS) worked equally well in differentiating these species. Consistent with these findings, Zhang et al. [15] used four genetic loci, such as rDNA ITS and the partial genes *CAL*, translation elongation factor-1 (*TEF1*), and -*3* (*TEF3*), to investigate the phylogeny of 99 clinical and 36 environmental strains belonging to the main pathogenic *Sporothrix* clade species (*S. schenckii sensu stricto*, *S. brasiliensis*, *S. globosa*, and *S. luriei*). Multilocus sequence data were compared with amplified fragment length polymorphism (AFLP) genotyping. The four species of the clade were recognized, and the clade comprised nine subclusters. The combined tree based on *CAL*, *TEF-1*, and *TEF-3* of 135 strains had a topology similar to that of the ITS tree. However, the authors noticed that the genes analyzed differed in PCR performance, whereas *CAL* showed a small intraspecific variability, which makes *CAL* an optimal diagnostic marker [15]. Accordingly, Rodrigues et al. [23] using *CAL* gene sequences identified specific PCR-based markers for clinically relevant members of the *Sporothrix* genus (*S. brasiliensis*, *S. schenckii*, *S. globosa*, *S. mexicana*, and *Sporothrix pallida*) and its relative *Ophiostoma* genus (*O. stenoceras*). The assay proved to be effective in identifying etiological agents of sporotrichosis, detecting *Sporothrix* DNA in tissues of infected animals, and using fungal conidia as a source of genomic DNA for PCR. Compared to PCR sequencing or other available molecular assays for *S. schenckii* complex identification [17], the PCR assay described in that study [23] yielded reliable results in a more rapid and less expensive manner. To give an example (Figure 1), we generated a phylogenetic tree with partial *CAL* gene sequences from the *Sporothrix* complex clinical clade isolates retrieved from GenBank (http://www.ncbi.nlm.nih.gov/BLAST, accessed on 1 December 2021). Sequence alignment using MEGA7 software (https://www.megasoftware.net, accessed on 1 December 2021) resulted in a tree showing that *S. schenckii sensu stricto* strains (ATCC 10268, UTHSC 04-1064, and CBS 359.36T) clustered separately from *S. brasiliensis* (Ss56, CBS132986, and UNIFESP Ss99), *S. globosa* (FHJU16, CBS 132925, and S10M), or *S*. *luriei* (ATCC 18616T) strains.

Recently, searching for faster and easier PCR-based methods culminated in the development of a multiplex real-time PCR assay by Zhang et al. [24], who designed primers and probes to target the *CAL* gene of clinically relevant *Sporothrix* species, such as *S. globosa*, *S. schenckii sensu stricto*, and *S. brasiliensis*. The diagnostic performance of the novel assay was evaluated in comparison with culture and species-specific PCR. Regarding clinical samples, the positive detection rates by culture, species-specific PCR, and the multiplex real-time PCR assay were 87.9% (29/33), 39.4% (13/33), and 93.9% (31/33), respectively. Regarding spiked samples, the positive detection rates were both 100% for *S. schenckii sensu stricto* and *S. brasiliensis*. Based on these findings, the authors claimed that the assay might transform into a new commercial kit for the diagnosis of infections caused by these clinically relevant *Sporothrix* species. Concomitantly, Hayashi et al. [25] explored the diagnostic value of a *Sporothrix*-specific nested PCR assay for diagnosing cutaneous sporotrichosis from formalin-fixed and paraffin-embedded (FFPE) tissues. The authors found that all (100%) of 52 samples from patients with a positive culture result tested positive by PCR, whereas 58 (98.3) of 59 samples from patients in the control group tested negative by PCR. Thus, the assay showed an excellent sensitivity (100%) and specificity (98.7%). Finally, de Carvalho et al. [26] used an in silico screening strategy of virtually generated AFLP fingerprints using whole-genome *Sporothrix* sequences, which resulted in six primer pair combinations to be tested in vitro. Three combinations provided the best diversity indices and lowest error rates. In general, the DNA fingerprint assay described in that study enabled the authors to assess the degree of intraspecific variability among pathogenic *Sporothrix* species, providing insights into their ecology and evolution [26].

### 2.3. Matrix-Assisted Laser Desorption Ionization Time-of-Flight-Based Methods

Despite still depending on fungal culture (which, as known, takes several days to yield visible growth), matrix-assisted laser desorption ionization time-of-flight (MALDI-TOF) mass spectrometry has become in many clinical microbiology laboratories a powerful and indispensable tool for the etiological diagnosis of filamentous fungal infections [27,28], including *S. schenckii* infections. In this context, Oliveira et al. [29] reported on the MALDI-TOF MS-based identification of 70 environmental and clinical isolates of the *Sporothrix* complex, which had previously been characterized by morphology (see above for fungus’s typical features). The authors adopted a two-step approach that has become a standard for studies dealing with the MALDI-TOF MS methods’ development for diagnostic applications. First, spectra from six isolates (all reference strains) were used to construct a reference database according to minimal adjustments in the manufacturer’s guidelines. Second, the database was challenged with spectra from remaining 64 isolates (all from different culture collections). Thus, the MALDI-TOF MS method distinguishing strains of *S. brasiliensis*, *S. globosa*, *S. mexicana*, *S. schenckii*, *S. luriei*, and *S. pallida* allowed the authors to identify all clinical and environmental isolates at the species level. This was in accordance with partial *CAL* gene sequence analyses performed on the same isolates [29].

Similarly, but more recently [30], MALDI-TOF MS analysis based on an in-house database enriched with reference spectra from *S.* *schenckii* complex was used to identify *S. brasiliensis* isolated from an immunocompetent patient’s subconjunctival infiltrative lesion. The fungus grown on SDA was identified as *Sporothrix* species by morphology (see above for fungus’s typical features), whereas its MALDI spectrum profile matched that of *S. brasiliensis* [30].

Taken together, these findings suggest the potential of complementary (self-constructed) in-house databases that, to date, support the MALDI-TOF MS-based fungal identification at least in large clinical microbiology laboratories [31]. Further standardization efforts are required before this approach becomes universally affordable in the routine clinical practice.

## 3. Genome and Dimorphism

Despite being evolutionarily divergent species, *S. schenckii sensu stricto* (hereafter referred to as *S. schenckii*) and *S. brasiliensis* are two (thermally dimorphic) fungal pathogens phenotypically similar to the human/animal pathogenic Onygenales (e.g., *Blastomyces dermatitidis*) but phylogenetically closely related to plant associated Sordariomycetes (e.g., *Grosmannia clavigera*). A comparative genomic analysis of the two *Sporothrix* species with the tree-pathogenic fungus *G. clavigera* showed *S. schenckii* and *S. brasiliensis* genomes with a G + C content being one of the highest within Ascomycota (62% versus 53.4% in *G. clavigera*) [14]. Of 4788 genes found in all three species’ genomes, 2001 were *Sporothrix*-restricted genes, whereas transport or metabolism genes showed a higher conservation degree than those from other Ascomycota. Remarkably, comparing *S. schenckii* and *S. brasiliensis* with fungal species from other Sordariomycetes or dimorphic fungal pathogens (Onygenales) unveiled the expansion of some protein domains and the lack of (polysaccharide lyase) genes associated with plant biomass decay [14]. According to the authors’ opinion [14], these findings suggest a unique ecological shift in the *Sporothrix* lineage, which allowed *S. schenckii* or *S. brasiliensis* (close relatives of plant associated Sordariomycetes) to adapt to mammalian parasitism. In this context, dimorphism would be a congruent evolution case, reinforcing the role of this morphogenetic shift into fungal pathogenesis [14].

Very recently, comparative studies extended our knowledge on phylogenomics and/or genomic evolution, as well as on pathogenicity/virulence-associated genes, of pathogenic *Sporothrix* species [32,33]. When showing that *Sporothrix* species are closely related to *Ophiostoma* species, one study using a gene family evolutionary analysis revealed that virulence, stress response, protease, cell wall composition, or transporter genes were expanded in *Sporothrix* species, supporting the genomic evolution of pathogenic *Sporothrix* species from *Ophiostoma* species [32]. Another study revealed that *S. brasiliensis*, *S. schenckii*, *S. globosa*, and *S. luriei* had significantly smaller genome sizes than other environmental and clinical *Sporothrix* species. This contraction mostly involved the genes associated with plant biomass decay such as CAZyme (Carbohydrate Active enZyme) or peptidase encoding genes, as well as the genes associated with loss of pathogenicity and reduced virulence [33]. This might explain not only an adaptive shift of *Sporothrix* species from a saprobic life in plant materials to a pathogenic life in mammals but also an increase in their pathogenicity during the evolution [33]. Until now, there is no known sexual stage in *Sporothrix* species [34], despite the presence of a set of mating or pheromone-response protein-encoding genes in their genomes [35].

As for other thermally dimorphic fungi [34], dimorphism in its strictest sense involves the ability of a fungus such as *Sporothrix* species to generate two types of vegetative cells, i.e., those that are either hyphal or yeast in morphology, which correspond to the environmental (observable at 30 °C) and the host (observable at 37 °C) form, respectively. In the first form, hyaline, septate hyphal cells produce several morphologically different asexual conidia, some of which are hyaline, oval or elongate, and originate individually or in small clusters (i.e., with a flower-like arrangement alongside the hyphae) from sympodial conidiogenous cells. Alternatively, conidia can be thick-walled, darkly pigmented, and usually produced individually. In the second form, yeast cells are round, oval, or lengthened (hence termed “cigar-shaped”) and divide by budding. While dimorphism is essential for virulence in the mammalian host [34], different *Sporothrix* species may present different levels of pathogenicity, which in turn depend on the interaction of the fungus with the innate and adaptive immune response cells through which the mammalian host controls and/or eradicates fungal pathogens [10]. Consistent with the ability of different innate immune receptors to recognize *Sporothrix* species [10], Martínez-Álvarez et al. [6] using conidia, yeast-like cells, and germlings of *S. schenckii* and *S. brasiliensis* showed that the two *Sporothrix* species differed regarding the ability to elicit cytokine production by human peripheral blood mononuclear cells (PBMCs). Three *S. schenckii* morphologies elicited higher levels of pro-inflammatory cytokines than *S. brasiliensis* whereas *S. brasiliensis* yeast-like cells and germlings elicited a higher level of the anti-inflammatory cytokine IL-10, perhaps accounting for the apparently lower pathogenicity of *S. schenckii* compared to *S. brasiliensis*. Additionally, dectin-1 was a key receptor for the *S. schenckii* elicited cytokine production whereas was dispensable for the cytokine production elicited by *S. brasiliensis* germlings [6].

To elucidate the transcriptional networks involved in the *S. schenckii* morphological (yeast-to-hypha) shift, Giosa et al. [36] used RNA-seq data to identify 17,307 genes, of which 11,217 and 6090 were classified, respectively, as protein-encoding genes or non-coding RNAs (ncRNAs). Comparative gene expression analysis revealed differential gene expression between yeast and hyphal cells that regarded not only 8795 genes but also antisense ncRNAs overlapping neighboring protein-encoding genes [36]. Thus, ncRNA would contribute to protein transcription within the complex system of molecular signaling pathways leading to the *S. schenckii* yeast-to-hypha shift. Furthermore, a transcriptome sequencing analysis of 48 h induced yeast and mycelial *S. schenckii* stages allowed Zheng et al. [37] to map the signaling pathways associated with the fungus’ morphological switch. This analysis identified G-protein signaling systems, Ras, and MAPK cascades as potential molecular mechanisms to control the *S. schenckii* dimorphic transition.

## 4. Cell Wall and Surface Components

According to well-established fungal pathogenesis models, the cell wall is the place where pathogen-associated molecular patterns interact with pattern recognition receptors (PRRs) on innate immune cells. One of these PRRs is dectin-1. The study by Martínez-Álvarez et al. [6] which investigated the *Sporothrix* cell wall components’ interaction with human PBMCs showed that *S. schenckii sensu stricto* and *S. brasiliensis* share a similar cell wall composition but display morphology-dependent changes. Interestingly, in *S. schenckii sensu stricto*, chitin (i.e., the N-acetylglucosamine basic unit) content was significantly different across the three morphologies (conidia, yeast-like cells, and germlings) studied by the authors. Conversely, in *S. brasiliensis*, β-glucan (i.e., the glucose basic unit) content was significantly lower in yeast-like cells compared to germlings [6]. In both *S. schenckii* and *S. brasiliensis*, according to ultrastructural data by Lopes-Bezerra et al. [38], a bi-layered cell wall structure during the yeast phase includes an external microfibrillar layer and an inner electron-dense layer. However, the *S. brasiliensis* cell wall layers were found to be thicker than those of *S. schenckii* were, and this finding correlated with increased contents of chitin and rhamnose cell wall components [21]. Unlike other dimorphic fungi, *Sporothrix* species are lacking the α-glucan component in their cell wall. Interestingly, in the same study [38], Lopes-Bezerra et al. identified glycogen α-particles in the cytoplasm close to the budding pole of yeast cells, which may be a source of glucose for cell wall enzymes.

In addition to β-glucans and chitin, the *S. schenckii* complex cell wall contains a glycoconjugate composed of rhamnose, mannose, glucuronic acid, and proteins, named peptidorhamnomannan (PMR). This cell wall component harbors *Sporothrix*-specific immunogenic epitopes [39,40] and, along with the 70 kDa glycoprotein (Gp70) located on the yeast phase surface (gp70), is involved in the host response immunomodulation [40]. Highly virulent *S. brasiliensis* clinical isolates tested by Castro et al. [41] in a murine subcutaneous model of sporotrichosis displayed reduced levels of Gp70, underlining the importance of this antigen (and adhesin) in the *S. schenckii* complex pathogenicity. While the PRM carbohydrate moiety has been extensively studied, only recently García-Carnero et al. [42] explored the PMR protein core. Of 325 proteins identified, the chaperonin GroEL/Hsp60 and the uncharacterized protein Pap1 were further investigated. The authors showed that both proteins could bind extracellular matrix proteins and contribute to the *S. schenckii* virulence. When *Galleria mellonella* larvae were inoculated with *S. schenckii* yeast-like cells pre-incubated with anti-rHsp60 or anti-rPap1 antibodies, larvae displayed increased survival rates compared to control groups [42].

Similar to other pathogenic fungi, *Sporothrix* species produce dihydroxynaphthalene-melanin (DHN-melanin) in conidia and yeast cells as well as L-dihydroxyphenylalanine (L-DOPA), which may enhance melanin production on both these structures and hyphae. To corroborate the role of melanin in the virulence of the *S. schenckii* complex, Almeida-Paes et al. [43] showed that *S. brasiliensis*, *S. schenckii sensu stricto*, and *S. globosa* can produce a melanoid pigment, named pyomelanin, in the presence of tyrosine, in both mycelial and yeast stages. Thus, pyomelanin-producing fungal cells were more resistant to nitrogen-derived oxidants and to UV light. Using a murine model of disseminated sporotrichosis, Texeira et al. [44] compared the virulence of *S. schenckii* yeast cells after they had been cultured on two media differing in their brain (and heart) infusion’s proportions. Such a difference allowed *S. schenckii* to modulate melanin expression on the surface of yeast cells and, consequently, virulence in mice. Interestingly, yeast cells grown in a brain-infusion poor medium supplemented with L-DOPA recovered their virulence, underlining rich brain-infusion medium’s phenolic compounds as a substrate for the melanin biosynthesis pathway.

In *S. brasiliensis*, extracellular vesicles (EVs), i.e., lipid bilayers exhibiting an array of biological properties, may aid the fungus to infect the host. In one study, Ikeda et al. [45] used *S. brasiliensis* EVs to modulate dendritic cells (DCs) and to control the infection in vivo. The study showed that EVs induced an increase in the phagocytic index and fungal burden in DCs, whereas BALB/c mice inoculated with a high concentration of EVs before subcutaneous infection showed an increase in the fungal burden and lesion diameter at 21 days after infection. In another study [46], yeast proteomic profiling of *S. brasiliensis*, *S. schenckii*, and *S. globosa* allowed to identify 247 proteins, of which 137 as differentially expressed. Most of differences between *S. brasiliensis* and *S. schenckii* or *S. globosa* regarded the amino acid metabolism and cell wall remodeling, along with downregulation of glycolytic enzymes, possibly explaining a more virulent behavior of *S. brasiliensis* compared to *S. schenckii* and *S. globosa* [46]. Finally, Ortega et al. [47] showed that *S. brasiliensis* is more resistant to different oxidants, such as H_2_O_2_ and menadione, than *S. schenckii*, perhaps because of mutations found in the *S. brasiliensis* Hog1 stress activated protein kinase. These mutations would allow *S. brasiliensis* to better adapt to the toxic reactive oxygen species generated by the host’s immune system.

## 5. Biofilm and Antifungal Resistance

In a very recent analysis of biofilm formation in the *S. schenckii* complex, Sánchez-Herrera et al. [48] tried to model the biofilm development stages that, similar to other fungal species, include adsorption, adhesion, microcolony formation, maturation, and dispersion. In the last stage, fungal cells depart biofilms to disseminate infectious foci to distant body sites. As in other fungal biofilms, extracellular matrix (ECM) in the *S. schenckii* biofilm contains mannose-rich glycoproteins, carbohydrates, lipids, and nucleic acids. However, *S. schenckii* biofilm’s ECM has greater carbohydrate and less protein amounts compared to those in the ECM from biofilms formed by *Candida* species (*C. albicans* and *C. parapsilosis*), which are the most common pathogenic fungal species in clinical settings [49].

Consistent with previous studies [49], a substantial presence of (extracellular) DNA in the *S. schenckii* biofilm ECM may not only contribute to the biofilm structure integrity but also to the biofilm-associated antifungal resistance in *S. schenckii* infections [48]. In this context, it is noteworthy that clinical isolates of *Sporothrix* species have increasingly been reported to be resistant to amphotericin B (AMB), azoles, and echinocandins [50,51,52]. The study by Rodrigues et al. [53], determining the minimum inhibitory concentration (MIC) values for AMB, fluconazole (FLZ), itraconazole (ITZ), voriconazole (VRZ), posaconazole (PCZ), flucytosine (5FC), and caspofungin (CSF) of four pathogenic species (*S. schenckii sensu stricto*, *S. brasiliensis*, *S. globosa*, and *S. luriei*), showed that AMB, 5FC, CSF had no antifungal activity against any *Sporothrix* species. Conversely, ITZ and PCZ were moderately effective against *S. schenckii sensu stricto* and *S. brasiliensis*. Interestingly, studying a panel of genetically diverse clinical *Sporothrix* isolates (from cutaneous-fixed, cutaneous-lymphatic, or disseminated disease cases) revealed the presence of genotypes (haplotypes) within each (clade of) *Sporothrix* species that were linked to the antifungal-susceptibility profiles shown by the isolates from each species [53].

In 2018, Brilhante et al. [54] investigated the ability of *S. schenckii* complex isolates to form biofilms in vitro as well as the growth kinetics, morphology, and antifungal susceptibility of these biofilms. The authors assessed the effects of exposure to antifungal drugs (AMB, CSF, FLZ, KCZ, ITZ, and VRZ) at MIC against planktonic cells as well as at 10 × MIC and 50 × MIC against biofilm cells. When confirming a strong biofilm formation by all *S. brasiliensis* (*n* = 10), *S. schenckii sensu stricto* (*n* = 2), *S. globosa* (*n* = 2), and *S. mexicana* (*n* = 4) isolates, Brilhante et al. [54] also found that biofilms formed by these isolates displayed a dense network of hyphae and conidia, which were immersed in a biofilm ECM endowed with water channels. As for planktonic MICs, biofilm MICs had values that differed across the species of the *S. schenckii* complex. Only for some antifungal drugs, exposure of *S. schenckii* biofilms to planktonic MIC values allowed to observe anti-biofilm activity, which yet was more evident at 50 × MIC values [54].

As recently reviewed [51], melanin (DHN-melanin, L-DOPA melanin, and pyomelanin) production, genetic diversity, and cytochrome P450 mutations are possible mechanisms of antifungal resistance in *Sporothrix* species. While melanin has been associated with lower AMB susceptibility and with protection against the effects of terbinafine (which may be used instead of AMB for sporotrichosis therapy), chromosomal polymorphism, amplifying antifungal resistance genes, may help *Sporothrix* species to overcome the selective pressure exerted by antifungal drugs [51]. However, this mechanism contributes to increase the environment adaptation of the fungus and/or the emergence of resistant *Sporothrix* isolates. Additionally, in silico analysis performed by Matowane et al. [55] showed that mutations in the P450 monooxygenase CYP51 (the main target of azole antifungal drugs), involving amino acids located at the channel entrance to the enzyme’s active, can be associated with ketoconazole resistance. This may be the reason why ketoconazole, unlike ITZ (the first-choice antifungal drug for sporotrichosis therapy), appears to be ineffective against sporotrichosis.

According to recently established epidemiological cut-off values [56], non-wild-type isolates (those with acquired resistance mechanisms to AMB, ITZ, terbinafine, and VRZ) have been identified across the species of the *Sporothrix schenckii* complex [51]. In this context, it should be noted that studies’ authors [51,52] tested their *Sporothrix* isolates for antifungal susceptibility in vitro using the Clinical and Laboratory Standards Institute (CLSI) reference methods [57,58]. It is still debated whether in vitro antifungal susceptibility testing should be performed on isolates in the mycelial or yeast phase [59]. However, it is noteworthy that in Bao et al.’s study [50], a CLSI-adapted method, the Sensititre™ YeastOne™ assay, was used to test mycelium phase and yeast phase susceptibility of *S. globosa* isolates to echinocandins (anidulafungin, micafungin, and CSF), 5FC, azoles (PCZ, VRZ, ITZ, FLZ), and AMB. The authors found that ITZ was the most active antifungal drug against *S. globosa*, whereas the yeast phase of the same isolate was more susceptible than that of the mycelium phase, leading to consider the lower content of melanin present in the fungus’ yeast phase after repeated cultures as a possible explanation [50].

## 6. Perspectives

The threat of emerging and re-emerging pathogenic *Sporothrix* species has represented [60] and so far, represents [61] a continuous stimulus to increase our knowledge on the epidemiology, transmission, diagnosis, and treatment of human and feline sporotrichosis. Regarding *S. brasiliensis*, a one-health approach aimed to optimize health outcomes, through collaborative efforts on the inter-relationships among humans, animals, plants, and the environment [62], should be undertaken to contrast the limited awareness of this fungal pathogen, especially in areas with low occurrence of cat-to-human transmitted sporotrichosis. This would imply a multidisciplinary cooperation by veterinarians, physicians, epidemiologists, microbiologists, and environmental scientists to control the occurrence of outbreaks in workers engaged with high-risk activities or of epidemics related to a common source of infection.

From the diagnostic standpoint, elucidating the fine structure of the surface in both the morphological stages of *S. schenckii* complex as well as the genome sequences of single species within the complex will permit to develop new identification or molecular typing tools that will improve the specificity of human or feline sporotrichosis diagnosis. Meanwhile, refinement of existing molecular laboratory methods is expected to embrace the possibility of targeting both virulence and antifungal resistance of *Sporothrix* species, two features thought to cause the selection and clonal expansion of resistant isolates during epidemics. From the therapeutic standpoint, emergence of ITZ-resistant *S. schenckii* complex isolates has prompted the research on new antifungal compounds such as essential oils. In one very recent study [63], the essential oil of rosemary (*Rosmarinus officinalis* Linn.) proved to protect rats from the fungal spread to systemic organs, thus providing new options for treatment of sporotrichosis.

In summary, great future advances in this important fungal topic will make the prospect of significantly reducing the burden of sporotrichosis for human and animal health a not-so-distant reality for us.
